# Odonto-sinusal influences

**Published:** 2015

**Authors:** MR Zamfir

**Affiliations:** * Morphology and Anatomy Department, “Carol Davila” University of Medicine and Pharmacy

**Keywords:** maxillary sinus, sinusitis, defense mechanism, inflammation, pulpolitis

## Abstract

There is a wide documentary material regarding the upper dental disease influencing the state of the maxillary sinus. This article tries to show that the relation is a two-way one.

As it is known, the vicinity between the maxillary sinus and the oral cavity, biunivocal answer to the pathological factors.

The premolars and upper molars roots are open in the lateral maxillary zone, nearby, or inside the maxillary sinus under the mucosa penetrating the cortical bone of the floor.

There is a layer of spongious bone under the cortical bone, whose height is variable: well represented in the edentulous maxilla and not so well represented in the edentulous areas (areas become like this during the preprosthetic extraction). There are situations in which the pathological pulp chamber process grows, after the death of the pulp, which reaches the apical bone and destroys it until the sinus of floor [**1**-**5**].

The cancellous bone tissue located at the maxillary lateral is well vascularized. Arterial circulation is achieved by branches of the maxillary artery: posterior superior alveolar arteries, infraorbital artery, ascending palatine artery and the sphenopalatine artery. However, in certain situations, such as the occurrence of inflammatory lasting processes (chronic sinusitis train), circulatory changes occur leading to bone resorption [**7**]. Sometimes, together with the bone resorption, a decrease in the amount of bone marrow occurs, which involves a problem of blood cells contribution.

The tooth loss from the side accentuates the process. The decrease of the vascular support tends to be compensated by the contribution of the blood from the periosteum, which, however, is insufficient.

Besides, bone remodeling occurs, together with the circulatory deficits points, remaining in the body of the tooth pulp.

In addition, in chronic rhinogenic sinusitis, which occurs after an acute sinusitis, the discreet clinical symptoms sometimes mask the local evolution in most cases. Unilateral rhinorrhea usually occurs, the feeling of clogged nostril and intermittent pain is also present especially in the morning and the growth in the ostia drainage blockage (sinus secretions remain blocked) [**4**]. Sometimes, there is a tension in the genian and infraorbital areas, which is accentuated by the declining of the head position. Cacosmia is permanent and subjective.

As a result of this evolution, chronic maxillary sinusitis is sometimes ignored until late stages - time which means years that have passed since the onset.

Sinusal mucosa that typically has 0.3-0.8 mm, becomes hyperemia, edema and increases in thickness, being edematous (can grow until it fills the sinus cavity – (**Fig. 1**, **Fig. 2**), showing crusts and ulcerations, sometimes looking like polyps. Polyps can pass through the ostia in the middle meatus. The patient accuses pain in the palpation of the front wall of the affected sinus. The puncture of the inferior meatus shows pus [**4**].

**Fig. 1:**
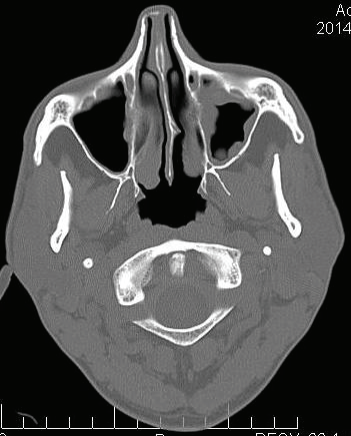
Chronic maxillary sinusitis

**Fig. 2:**
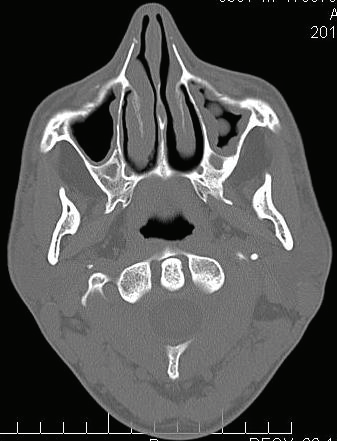
Maxillary sinus polyp

Radiological investigations found a unilateral sinus opacification. It highly marks the periphery and is less central. This is mainly due to the thickening of the mucosa [**8**].

Microbiology-mucosa shows a plasma cell inflammatory infiltrate. There are many polyps, and in more advanced stages, they comprise all the sinus polyps that can obstruct and block the drainage ostia. The drainage ostium appears as emphysema due to the process and sometimes the rebound effect. The microbial germs, which are commonly involved in sinusitis, are streptococcus pneumoniae, staphylococcus, and colibacillus, Klebsiela, proteus and piocianic [**6**].

All these explain why an inadequate medical treatment appears in the evolution around the affected sinus, osteitis and osteomyelitis. The inflammation surrounding the bone tissue has an effect on the appearance of the local hyperemia and a state of constant irritation to the surrounding area.

In the periapical tissue, the inflammation of the cortical bone appears very late due to its thickness and density. The capillary hyperemia with the transudation accumulation appears in the cancelled bone. The liquid flows gravitationally along the bone trabeculars [**9**].

At the dental apex level, which comes in the pulp, one or few arteries splint in the terminal arteriole. Sometimes, before splinting, the artery has a shortcut to the venous circulation. At the apex level, 2-3 veins leave the tooth and make an anastomosis with the blood vessels from the periodontal space or from the cancellous bone around.

Pulp vessels have very thin walls with very few muscle fibers. The existence of a shortcut permits the redistribution of the blood flow. Tooth pulp structure is affected by the increase of the pressure in the narrow tissue, a fact that in time produces irreversible metabolic changes. In the same time, a cellular answer appears with the growth of the inflammation level. The collagen hydrolysis produces the kinina, which makes the vasodilatation and increases the vascular permeability. The blood is redistributed in the arteriovenous shortcuts to the periphery. The oxygen contribution becomes smaller and the pulp is suffocated, a fact that increases the inflammation. Necrotic cells become calcified. The odontoblast stimulated by means from inside the pulp (not from the Thomes tubes) starts to produce secondary dentin. In case of death of the odontoblast, it can be replaced by the mesenchymal undifferentiated cells. Moreover, other times, they become odontoclasts and remodel the shape of the inner tooth. The pulp chamber is filled with pulpitis (**Fig. 3**) (accumulation of mineral tissue around an organic metrics). Sometimes, the root channels are also filled with mineral tissue. The whole tooth appears like a single structure, with the loss of the pulp chamber X-ray images.

**Fig. 3:**
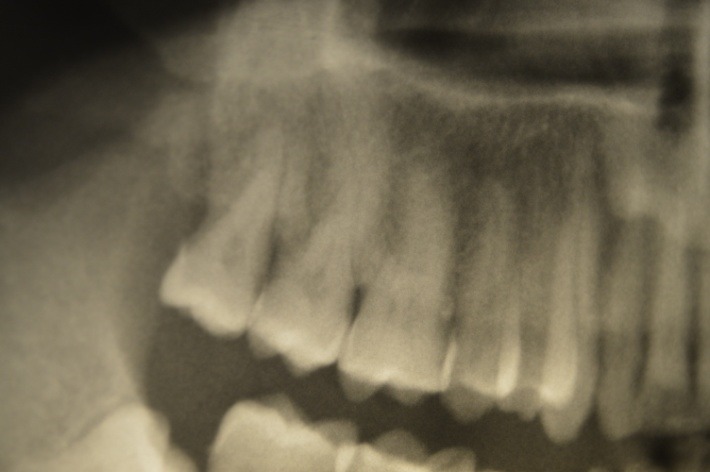
X-ray upper molars with pulp chamber modification

This evolution (appearance of the pulpitis) is not a general one. It only appears in few cases.

In a study of 31 cases of patients with ages between 16 and 60 years, all suffering from chronic rhinogenic sinusitis, only 5 patients showed signs of X-ray pulp chamber modifications. In addition, just one of them showed a complete pulp chamber image loosing. Even with such results, it is very probable that not just the apical inflammation influenced the sinusal evolution; also, the sinusal inflammation modified the upper tooth.
